# Poly[[μ_2_-1,4-bis­(imidazol-1-ylmethyl)­benzene](μ_4_-3,5,9,11-tetra­oxo-4,10-diaza­tetra­cyclo­[5.5.2.0^2,6^.0^8,12^]tetra­dec-13-ene-4,10-diido)disilver(I)]

**DOI:** 10.1107/S1600536812039669

**Published:** 2012-09-22

**Authors:** Yongmei Zhang

**Affiliations:** a School of Chemistry and Life Science, Anshan Normal University, Anshan, Liaoning 114000, People’s Republic of China

## Abstract

In the title complex, [Ag_2_(C_12_H_8_N_2_O_4_)(C_14_H_14_N_4_)]_*n*_, one Ag^I^ ion, lying on a twofold rotation axis, is coordinated by two N atoms from two 3,5,9,11-tetra­oxo-4,10-diaza­tetra­cyclo­[5.5.2.0^2,6^.0^8,12^]tetra­dec-13-ene-4,10-diide (*L*) ligands in a nearly linear arrangement. The other Ag^I^ ion, lying on an inversion center, is coordinated by two O atoms from two *L* ligands and two N atoms from two 1,4-bis­(imidazol-1-ylmeth­yl)benzene ligands in a distorted square-planar geometry. An additional Ag⋯Ag [3.0119 (3) Å] inter­action links the Ag^I^ ions into a chain along [010]. The two types of ligands have mirror symmetry and connect the Ag^I^ ions into a layer parallel to (100).

## Related literature

For the design and synthesis of coordination polymers, see: Liao *et al.* (2008 ▶); Song *et al.* (2012 ▶); Wang *et al.* (2009 ▶). For the van der Waals radius of the Ag atom, see: Bondi (1964 ▶).
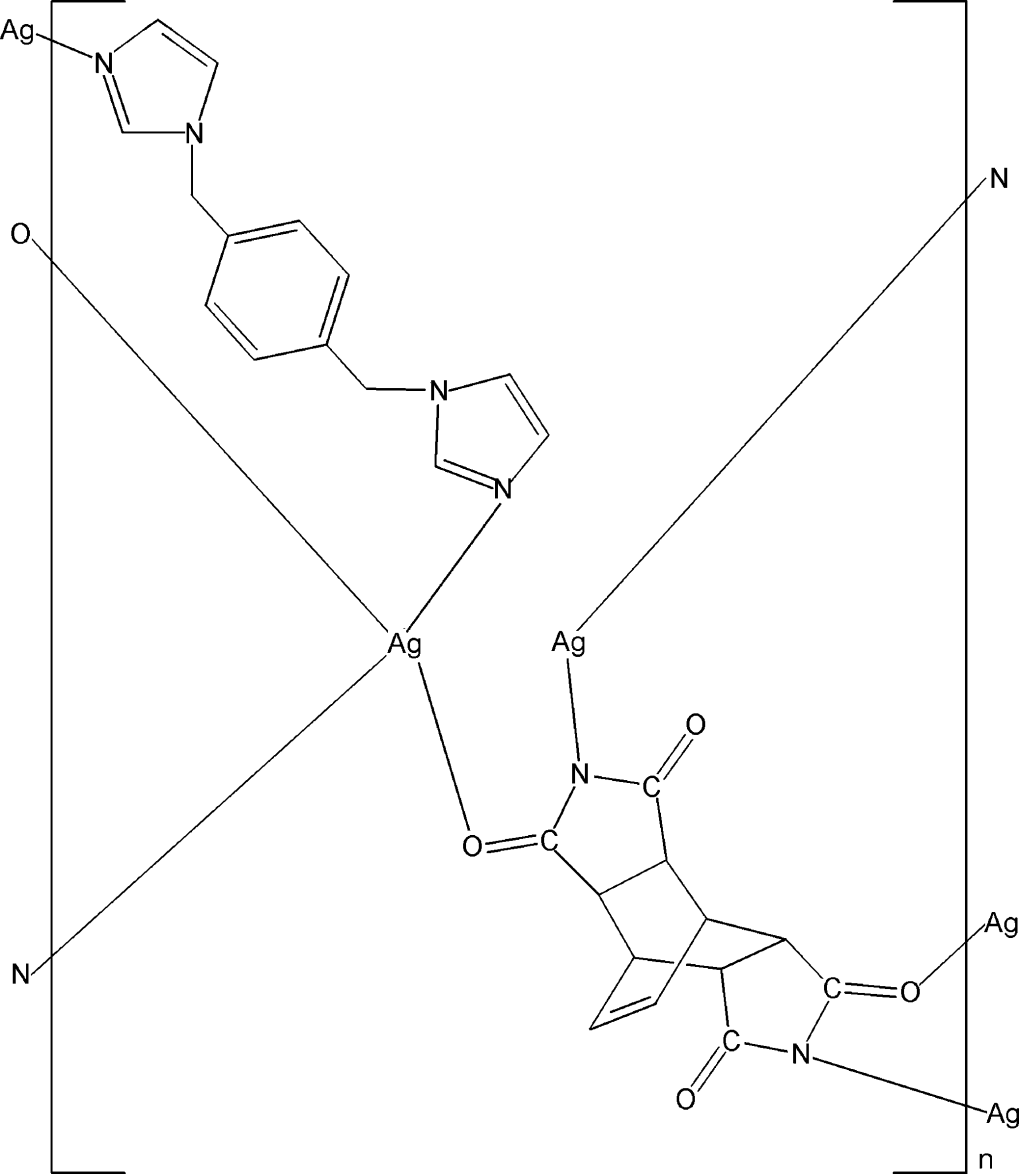


## Experimental

### 

#### Crystal data

[Ag_2_(C_12_H_8_N_2_O_4_)(C_14_H_14_N_4_)]
*M* *_r_* = 698.24
Orthorhombic, 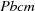

*a* = 10.1480 (11) Å
*b* = 11.0016 (11) Å
*c* = 21.183 (2) Å
*V* = 2365.0 (4) Å^3^
*Z* = 4
Mo *K*α radiation
μ = 1.71 mm^−1^
*T* = 296 K
0.27 × 0.21 × 0.17 mm

#### Data collection

Bruker APEXII CCD diffractometer
Absorption correction: multi-scan (*SADABS*; Bruker, 2001 ▶) *T*_min_ = 0.656, *T*_max_ = 0.760
12177 measured reflections
2402 independent reflections
1454 reflections with *I* > 2σ(*I*)
*R*_int_ = 0.056

#### Refinement

*R*[*F* ^2^ > 2σ(*F* ^2^)] = 0.034
*wR*(*F* ^2^) = 0.094
*S* = 1.00
2402 reflections
180 parameters
H-atom parameters constrained
Δρ_max_ = 0.58 e Å^−3^
Δρ_min_ = −0.59 e Å^−3^

### 

Data collection: *APEX2* (Bruker, 2007 ▶); cell refinement: *SAINT-Plus* (Bruker, 2007 ▶); data reduction: *SAINT-Plus*; program(s) used to solve structure: *SHELXS97* (Sheldrick, 2008 ▶); program(s) used to refine structure: *SHELXL97* (Sheldrick, 2008 ▶); molecular graphics: *XP* in *SHELXTL* (Sheldrick, 2008 ▶) and *DIAMOND* (Brandenburg, 1999 ▶); software used to prepare material for publication: *SHELXTL* .

## Supplementary Material

Crystal structure: contains datablock(s) global, I. DOI: 10.1107/S1600536812039669/ng5293sup1.cif

Structure factors: contains datablock(s) I. DOI: 10.1107/S1600536812039669/ng5293Isup2.hkl

Additional supplementary materials:  crystallographic information; 3D view; checkCIF report

## Figures and Tables

**Table 1:** Selected bond lengths (Å)

Ag1—N1	2.078 (4)
Ag2—N2	2.141 (4)
Ag2—O2	2.693 (3)

**Fig. 1.**
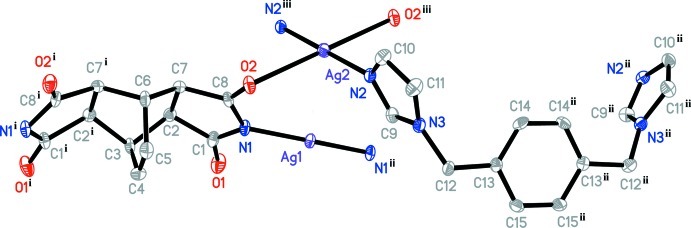
The asymmetric unit of the title compound. Displacement ellipsoids are drawn at the 30% probability level. [Symmetry codes: (i) x, y, 1/2 - z; (ii) x, y, 3/2 - z; (iii) -x, -y, 1 - z.]

**Fig. 2.**
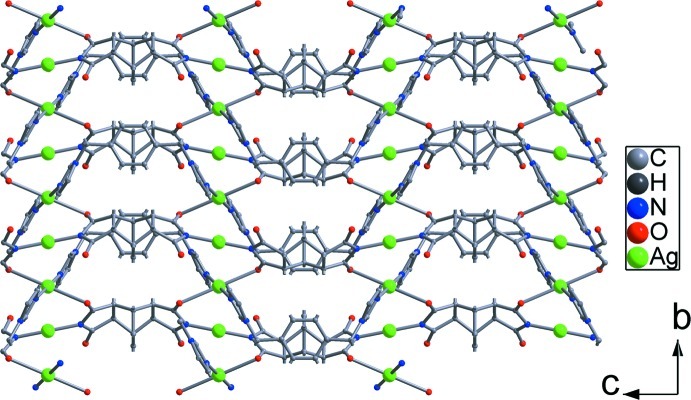
View of the layer structure of the title compound.

## supplementary crystallographic information

### Comment

Metal-organic frameworks (MOFs) are an emerging class of periodic crystalline solid-state materials constructed from metal ions or polynuclear metal-oxygen clusters and multidentate organic ligands. Recently, chemists have devoted themselves to the design and syntheses of coordination polymers, not only owing to their potential applications in the realm of gas adsorption and separation, catalysis, magnetism, luminescence and host–guest chemistry and *etc*, but also for their aesthetic and often complicated architectures and topologies (Liao *et al.*, 2008; Song *et al.*, 2012; Wang *et al.*, 2009). Bicyclo[2.2.2]oct-7-ene-2,3,5,6-tetracarboxydiimide (H_2_*L*), prepared by the ammonolysis of bicyclo[2.2.2]oct-7-ene-2,3,5,6-tetracarboxylic dianhydride, contains two kinds of possible coordination donors (N and O) to ligate metal atoms. Herein, we report a coordination polymer by simultaneous use of the H_2_*L* ligand and a neutral ligand, 1,4-bis(imidazol-l-ylmethyl)benzene.

As shown in Fig. 1 and Table 1, the title complex contains two crystallographically unique Ag^I^ ions. The Ag1 atom, lying on a twofold rotation axis, is coordinated by two N atoms from two 3,5,9,11-tetraoxo-4,10-diazatetracyclo [5.5.2.0^2,6^.0^8,12^]tetradec-13-ene-4,10-diido (*L*_1_) ligands in a nearly linear arrangement. The Ag2 atom, lying on an inversion center, is coordinated by two O atoms from two *L*_1_ ligands and two N atoms from two 1,4-bis(imidazol-1-ylmethyl)benzene (*L*_2_) ligands in a distorted square-planar geometry. The Ag···Ag separation [3.0119 (3) Å] is shorter than the sum of van der Waals radii for two silver atoms (3.44 Å) (Bondi, 1964), which indicates relatively strong argentophilicity. The *L*_1_ and *L*_2_ ligands, both have a mirror symmetry, connect the Ag^I^ ions into a layer structure parallel to (1 0 0) (Fig. 2).

### Experimental

A mixture of bicyclo[2,2,2]oct-7-ene-2,3,5,6-tetracarboxylic dianhydride (0.2 mmol, 0.050 g), 1,4-bis(imidazol-l-ylmethyl)benzene (0.2 mmol, 0.048 g), silver nitrate (0.4 mmol, 0.068 g) and H_2_O (25 ml) was stirred for ten minutes. Dilute ammonia was dropwised into the mixture until the mixture turned to transparent. Colorless block crystals of the title compound were isolated after evaporation of ammonia.

### Refinement

H atoms were positioned geometrically and refined as riding atoms, with C—H = 0.93 (aromatic), 0.98 (CH) and 0.97 (CH_2_) Å and with *U*_iso_(H) = 1.2*U*_eq_(C).

### Crystal data

**Table:**

[Ag_2_(C_12_H_8_N_2_O_4_)(C_14_H_14_N_4_)]	*F*(000) = 1384
*M**_r_* = 698.24	*D*_x_ = 1.961 Mg m^−^^3^
Orthorhombic, *P**b**c**m*	Mo *K*α radiation, λ = 0.71073 Å
Hall symbol: -P 2c 2b	Cell parameters from 3256 reflections
*a* = 10.1480 (11) Å	θ = 2.8–25.9°
*b* = 11.0016 (11) Å	µ = 1.71 mm^−^^1^
*c* = 21.183 (2) Å	*T* = 296 K
*V* = 2365.0 (4) Å^3^	Block, colorless
*Z* = 4	0.27 × 0.21 × 0.17 mm

### Data collection

**Table:**

Bruker APEXII CCD diffractometer	2402 independent reflections
Radiation source: fine-focus sealed tube	1454 reflections with *I* > 2σ(*I*)
Graphite monochromator	*R*_int_ = 0.056
φ and ω scans	θ_max_ = 26.0°, θ_min_ = 1.9°
Absorption correction: multi-scan (*SADABS*; Bruker, 2001)	*h* = −12→12
*T*_min_ = 0.656, *T*_max_ = 0.760	*k* = −6→13
12177 measured reflections	*l* = −26→24

### Refinement

**Table:**

Refinement on *F*^2^	Primary atom site location: structure-invariant direct methods
Least-squares matrix: full	Secondary atom site location: difference Fourier map
*R*[*F*^2^ > 2σ(*F*^2^)] = 0.034	Hydrogen site location: inferred from neighbouring sites
*wR*(*F*^2^) = 0.094	H-atom parameters constrained
*S* = 1.00	*w* = 1/[σ^2^(*F*_o_^2^) + (0.0389*P*)^2^ + 3.7137*P*] where *P* = (*F*_o_^2^ + 2*F*_c_^2^)/3
2402 reflections	(Δ/σ)_max_ < 0.001
180 parameters	Δρ_max_ = 0.58 e Å^−^^3^
0 restraints	Δρ_min_ = −0.59 e Å^−^^3^

### Special details

**Table:**

Geometry. All e.s.d.'s (except the e.s.d. in the dihedral angle between two l.s. planes) are estimated using the full covariance matrix. The cell e.s.d.'s are taken into account individually in the estimation of e.s.d.'s in distances, angles and torsion angles; correlations between e.s.d.'s in cell parameters are only used when they are defined by crystal symmetry. An approximate (isotropic) treatment of cell e.s.d.'s is used for estimating e.s.d.'s involving l.s. planes.
Refinement. Refinement of *F*^2^ against ALL reflections. The weighted *R*-factor *wR* and goodness of fit *S* are based on *F*^2^, conventional *R*-factors *R* are based on *F*, with *F* set to zero for negative *F*^2^. The threshold expression of *F*^2^ > σ(*F*^2^) is used only for calculating *R*-factors(gt) *etc*. and is not relevant to the choice of reflections for refinement. *R*-factors based on *F*^2^ are statistically about twice as large as those based on *F*, and *R*- factors based on ALL data will be even larger.

### Fractional atomic coordinates and isotropic or equivalent isotropic displacement parameters (Å^2^)

**Table:**

	*x*	*y*	*z*	*U*_iso_*/*U*_eq_	
C1	−0.2448 (5)	0.2484 (5)	0.3711 (2)	0.0310 (10)	
C2	−0.2537 (5)	0.1795 (4)	0.3080 (2)	0.0280 (11)	
H2	−0.3320	0.1274	0.3076	0.034*	
C3	−0.2545 (7)	0.2650 (6)	0.2500	0.0272 (16)	
H3	−0.3315	0.3188	0.2500	0.033*	
C4	−0.1301 (8)	0.3340 (7)	0.2500	0.0347 (18)	
H4	−0.1280	0.4185	0.2500	0.042*	
C5	−0.0208 (7)	0.2669 (7)	0.2500	0.0340 (19)	
H5	0.0633	0.3006	0.2500	0.041*	
C6	−0.0445 (7)	0.1334 (7)	0.2500	0.0281 (17)	
H6	0.0385	0.0879	0.2500	0.034*	
C7	−0.1291 (5)	0.1015 (4)	0.3074 (2)	0.0272 (11)	
H7	−0.1521	0.0150	0.3070	0.033*	
C8	−0.0604 (5)	0.1346 (5)	0.3690 (2)	0.0301 (12)	
C9	0.1964 (5)	0.1716 (5)	0.5666 (2)	0.0318 (13)	
H9	0.1268	0.2226	0.5772	0.038*	
C10	0.3079 (5)	0.0219 (5)	0.5312 (3)	0.0331 (13)	
H10	0.3296	−0.0512	0.5118	0.040*	
C11	0.3947 (5)	0.0980 (5)	0.5586 (3)	0.0352 (13)	
H11	0.4853	0.0874	0.5619	0.042*	
C12	0.3719 (5)	0.3029 (5)	0.6131 (2)	0.0289 (11)	
H12A	0.3208	0.3726	0.5994	0.035*	
H12B	0.4625	0.3164	0.6003	0.035*	
C13	0.3669 (4)	0.2965 (4)	0.6840 (2)	0.0236 (10)	
C14	0.3774 (5)	0.1894 (5)	0.7176 (2)	0.0308 (12)	
H14	0.3845	0.1161	0.6960	0.037*	
C15	0.3563 (5)	0.4041 (4)	0.7176 (2)	0.0308 (12)	
H15	0.3490	0.4774	0.6959	0.037*	
N1	−0.1358 (4)	0.2134 (4)	0.40402 (18)	0.0290 (10)	
N2	0.1828 (4)	0.0679 (4)	0.53608 (19)	0.0276 (10)	
N3	0.3223 (4)	0.1942 (4)	0.58061 (18)	0.0267 (9)	
O1	−0.3267 (4)	0.3211 (4)	0.38995 (17)	0.0445 (10)	
O2	0.0466 (3)	0.0946 (3)	0.38487 (16)	0.0346 (9)	
Ag1	−0.12096 (5)	0.2500	0.5000	0.02695 (15)	
Ag2	0.0000	0.0000	0.5000	0.03030 (16)	

### Atomic displacement parameters (Å^2^)

**Table:**

	*U*^11^	*U*^22^	*U*^33^	*U*^12^	*U*^13^	*U*^23^
C1	0.030 (2)	0.043 (3)	0.020 (2)	−0.001 (3)	0.005 (2)	−0.005 (3)
C2	0.028 (3)	0.030 (3)	0.026 (3)	−0.005 (2)	−0.001 (2)	0.000 (2)
C3	0.029 (4)	0.038 (5)	0.015 (3)	0.014 (4)	0.000	0.000
C4	0.042 (5)	0.027 (4)	0.036 (5)	−0.005 (4)	0.000	0.000
C5	0.030 (4)	0.046 (6)	0.026 (4)	−0.006 (4)	0.000	0.000
C6	0.030 (4)	0.038 (5)	0.017 (4)	0.008 (4)	0.000	0.000
C7	0.035 (3)	0.022 (3)	0.024 (3)	0.000 (2)	−0.001 (2)	0.000 (2)
C8	0.040 (3)	0.035 (3)	0.015 (3)	0.000 (3)	0.001 (2)	0.006 (2)
C9	0.030 (3)	0.035 (3)	0.031 (3)	0.002 (2)	−0.010 (2)	0.001 (2)
C10	0.033 (3)	0.035 (3)	0.032 (3)	−0.005 (2)	0.003 (2)	−0.007 (2)
C11	0.020 (3)	0.044 (3)	0.042 (3)	−0.004 (2)	0.000 (2)	−0.008 (3)
C12	0.033 (3)	0.029 (3)	0.024 (3)	−0.008 (2)	−0.008 (2)	0.001 (2)
C13	0.018 (2)	0.029 (3)	0.024 (2)	−0.002 (2)	−0.003 (2)	0.000 (2)
C14	0.038 (3)	0.023 (3)	0.031 (3)	−0.002 (2)	0.004 (2)	−0.011 (2)
C15	0.041 (3)	0.022 (3)	0.029 (3)	0.004 (2)	0.000 (2)	0.003 (2)
N1	0.032 (2)	0.040 (3)	0.015 (2)	0.0025 (19)	0.0010 (18)	−0.0032 (17)
N2	0.031 (2)	0.029 (2)	0.022 (2)	−0.0052 (19)	−0.0065 (18)	0.0016 (19)
N3	0.030 (2)	0.029 (2)	0.020 (2)	−0.0038 (19)	−0.0018 (19)	−0.0005 (19)
O1	0.036 (2)	0.065 (3)	0.033 (2)	0.016 (2)	0.0056 (18)	−0.014 (2)
O2	0.033 (2)	0.047 (2)	0.0235 (19)	0.0068 (18)	−0.0057 (16)	0.0033 (17)
Ag1	0.0308 (3)	0.0334 (3)	0.0167 (3)	0.000	0.000	−0.0039 (2)
Ag2	0.0293 (3)	0.0300 (3)	0.0316 (3)	−0.0038 (2)	−0.0104 (3)	0.0022 (3)

### Geometric parameters (Å, º)

**Table:**

C1—O1	1.221 (6)	C9—N3	1.334 (6)
C1—N1	1.363 (6)	C9—H9	0.9300
C1—C2	1.538 (6)	C10—C11	1.347 (7)
C2—C7	1.527 (7)	C10—N2	1.370 (6)
C2—C3	1.548 (6)	C10—H10	0.9300
C2—H2	0.9800	C11—N3	1.370 (6)
C3—C4	1.473 (10)	C11—H11	0.9300
C3—C2^i^	1.548 (6)	C12—N3	1.469 (6)
C3—H3	0.9800	C12—C13	1.505 (6)
C4—C5	1.333 (10)	C12—H12A	0.9700
C4—H4	0.9300	C12—H12B	0.9700
C5—C6	1.487 (9)	C13—C14	1.381 (7)
C5—H5	0.9300	C13—C15	1.385 (7)
C6—C7^i^	1.530 (6)	C14—C14^ii^	1.372 (10)
C6—C7	1.530 (6)	C14—H14	0.9300
C6—H6	0.9800	C15—C15^ii^	1.373 (10)
C7—C8	1.524 (7)	C15—H15	0.9300
C7—H7	0.9800	Ag1—N1	2.078 (4)
C8—O2	1.218 (6)	Ag2—N2	2.141 (4)
C8—N1	1.374 (6)	Ag2—O2	2.693 (3)
C9—N2	1.319 (6)	Ag1—Ag2	3.0119 (3)
			
O1—C1—N1	124.7 (4)	N2—C10—H10	124.9
O1—C1—C2	124.5 (5)	C10—C11—N3	106.0 (5)
N1—C1—C2	110.7 (4)	C10—C11—H11	127.0
C7—C2—C1	103.6 (4)	N3—C11—H11	127.0
C7—C2—C3	109.8 (4)	N3—C12—C13	114.7 (4)
C1—C2—C3	113.0 (4)	N3—C12—H12A	108.6
C7—C2—H2	110.1	C13—C12—H12A	108.6
C1—C2—H2	110.1	N3—C12—H12B	108.6
C3—C2—H2	110.1	C13—C12—H12B	108.6
C4—C3—C2^i^	108.0 (4)	H12A—C12—H12B	107.6
C4—C3—C2	108.0 (4)	C14—C13—C15	118.1 (5)
C2^i^—C3—C2	105.1 (5)	C14—C13—C12	123.5 (5)
C4—C3—H3	111.8	C15—C13—C12	118.4 (4)
C2^i^—C3—H3	111.8	C14^ii^—C14—C13	121.0 (3)
C2—C3—H3	111.8	C14^ii^—C14—H14	119.5
C5—C4—C3	115.3 (7)	C13—C14—H14	119.5
C5—C4—H4	122.3	C15^ii^—C15—C13	120.9 (3)
C3—C4—H4	122.3	C15^ii^—C15—H15	119.6
C4—C5—C6	114.3 (7)	C13—C15—H15	119.6
C4—C5—H5	122.8	C1—N1—C8	110.7 (4)
C6—C5—H5	122.8	C1—N1—Ag1	120.4 (3)
C5—C6—C7^i^	108.5 (4)	C8—N1—Ag1	127.6 (3)
C5—C6—C7	108.5 (4)	C9—N2—C10	105.0 (4)
C7^i^—C6—C7	105.4 (6)	C9—N2—Ag2	124.6 (3)
C5—C6—H6	111.4	C10—N2—Ag2	130.3 (3)
C7^i^—C6—H6	111.4	C9—N3—C11	107.1 (4)
C7—C6—H6	111.4	C9—N3—C12	125.8 (4)
C8—C7—C2	103.8 (4)	C11—N3—C12	127.2 (4)
C8—C7—C6	111.7 (4)	N1—Ag1—N1^iii^	171.7 (2)
C2—C7—C6	110.0 (4)	N1—Ag1—Ag2^iii^	101.91 (12)
C8—C7—H7	110.4	N1^iii^—Ag1—Ag2^iii^	81.53 (11)
C2—C7—H7	110.4	N1—Ag1—Ag2	81.53 (11)
C6—C7—H7	110.4	N1^iii^—Ag1—Ag2	101.91 (11)
O2—C8—N1	125.1 (5)	Ag2^iii^—Ag1—Ag2	131.898 (18)
O2—C8—C7	123.9 (5)	N2—Ag2—N2^iv^	180.00 (11)
N1—C8—C7	110.9 (4)	N2—Ag2—Ag1^iv^	88.01 (11)
N2—C9—N3	111.7 (5)	N2^iv^—Ag2—Ag1^iv^	91.99 (11)
N2—C9—H9	124.1	N2—Ag2—Ag1	91.99 (11)
N3—C9—H9	124.1	N2^iv^—Ag2—Ag1	88.01 (11)
C11—C10—N2	110.1 (5)	N2—Ag2—O2	92.08 (13)
C11—C10—H10	124.9	O2—Ag2—N2^iv^	87.92 (13)

Symmetry codes: (i) *x*, *y*, −*z*+1/2; (ii) *x*, *y*, −*z*+3/2; (iii) *x*, −*y*+1/2, −*z*+1; (iv) −*x*, −*y*, −*z*+1.
